# ON THE PRIVACY AND UTILITY PROPERTIES OF TRIPLE MATRIX-MASKING

**DOI:** 10.29012/jpc.674

**Published:** 2020-06

**Authors:** A. ADAM DING, GUANHONG MIAO, SAMUEL S. WU

**Affiliations:** Department of Mathematics, Northeastern University, Boston, MA; Department of Biostatistics, University of Florida, Gainesville, FL.; Department of Biostatistics, University of Florida, Gainesville, FL.

**Keywords:** Data masking, privacy protection, matrix masking

## Abstract

Privacy protection is an important requirement in many statistical studies. A recently proposed data collection method, triple matrix-masking, retains exact summary statistics without exposing the raw data at any point in the process. In this paper, we provide theoretical formulation and proofs showing that a modified version of the procedure is strong collection obfuscating: no party in the data collection process is able to gain knowledge of the individual level data, even with some partially masked data information in addition to the publicly published data. This provides a theoretical foundation for the usage of such a procedure to collect masked data that allows exact statistical inference for linear models, while preserving a well-defined notion of privacy protection for each individual participant in the study. This paper fits into a line of work tackling the problem of how to create useful synthetic data without having a trustworthy data aggregator. We achieve this by splitting the trust between two parties, the “masking service provider” and the “data collector.”

## 1. Introduction

In the digital age, vast amount of data becomes available for research. At the same time, there is increasing pressure to protect the privacy of study subjects when their data is used. For medical research, the Health Insurance Portability and Accountability Act of 1996 and subsequent rulings have imposed legal requirements for privacy protection on the collection and handling of health data. Among other things, basic privacy protection measures include the removal of all personal identifiers when releasing data for use. However, simply removing the personal identifier variables does not prevent possible identification of the individual from other variables. To prevent the identification of an individual record, researchers have shown that released data should be aggregated to satisfy privacy conditions such as k-anonymity [Sweeney, 2002], l-diversity [Machanavajjhala et al., 2007] and t-closeness [Li et al., 2007].

However, releasing data only at aggregated levels severely restricts its usefulness in many research studies. Alternatively, methods are designed to release obfuscated micro-data that allows for the usual statistical analysis while preserving the privacy at individual levels. Some examples of such obfuscated micro-data publishing are: noise addition [Brand, 2002], multiple imputation[Rubin, 1993, Drechsler and Reiter, 2010], information preserving statistical obfuscation [Burridge, 2003], random projection based perturbation [Liu et al., 2006], random orthogonal matrix masking [Ting et al., 2008]. Particularly, in the random orthogonal matrix masking scheme, a masked data set ***AX*** is published, where ***X*** denotes the data matrix of real responses and ***A*** is a random orthogonal matrix. The published data ***AX*** keeps the exact values for sufficient statistics of linear models, thus allowing exact statistical inference for many standard data analysis methods [Ting et al., 2008, Wu et al., 2017b] while protecting privacy by denying the user’s direct access to the raw data ***X***. While the above methods all protect the privacy of individual entries through publishing only the random perturbed micro-data, the privacy protection can be lost when multiple micro-data sets are combined from multiple inquires to the same database. Differential privacy is proposed to quantify the effectiveness of privacy protection of the random noise addition/perturbation schemes [Dwork et al., 2006, Dwork, 2006, Dwork and Naor, 2008] against multiple inquires to the database. Then the noise level can be adjusted to achieve a quantified tradeoff between inference efficacy and privacy preservation (measured by the differential privacy metric).

Traditionally, there is a trustworthy data collector/manager that collects raw data and ensure privacy protection by releasing the data sets with random perturbations. Such procedures however do not protect against attacks where an unscrupulous party has unauthorized access to the raw data set ***X*** kept by these centers. Such security breaks are becoming more common as shown by the recent well-publicized incidences involving hacking against databases at major retailers, banks and credit bureaus [Huffington Post, 2011, Reuters., 2015, 2017].

This paper fits into a line of work tackling the problem of how to create useful synthetic data without having a trustworthy data aggregator, and provides a theoretical study of the triple matrix-masking (TM^2^) procedure [Wu et al., 2017b] that does not assume such a trustworthy data collector/manager. The TM^2^ procedure is a multi-party collection and masking system that aims to collect and publish the random orthogonal masked data set ***AX***. We prove that, assuming no collusion between parties, no party learns more than the orbit of the data matrix under the action of the orthogonal group. More specifically, given the view of a particular party, let S be the set of data matrices that could possibly have resulted in that view. We show that S contains the full orbit of the data matrix and that given any prior on the data matrix, the party’s posterior is simply their prior restricted to S. We call data collection procedures with such properties as *strong obfuscating* since any extra information beyond ***AX*** available to a party does not help in further identifying the individual level data.

In the differential privacy literature, the issue of untrustworthy data collector can be dealt with using *local differential privacy* procedures [Kasiviswanathan et al., 2011], where noises are added to the individual data before passing to the data collector. The resulting synthetic data from differential privacy procedures, however, does not preserve exact statistics hence require special inference procedures designed to achieve optimal statistical inference Duchi et al. [2017]. Our TM^2^ procedure provides an alternative where the published masked data exactly preserve any statistics of the data that are preserved under the action of the orthogonal group. This provides an useful utility that exact statistical inference for linear models are preserved, thus standard linear statistical inference procedures can be applied directly on the resulting synthetic data from the TM^2^ procedure. On the other hand, the TM^2^ procedure is only for a one-shot collection of each individual’s data. When the individual data providers are sampled in multiple independent collections by different data collectors, differential privacy procedures can measure and limit the privacy leakage for the composition of the multiple collections. The TM^2^ procedure does not consider the privacy leakage for the composition of the multiple collections.

Section 2 describe the TM^2^ procedure and two new modifications to make it strong obfuscating. The theoretical analysis is provided in Section 3. Section 4 provides a summary and more detailed discussions for the relationship of the TM^2^ procedure to the differential privacy and multi party computation methods.

## 2. The Masked Data Collection Procedure TM^2^nd Its Modification

The privacy-preserving data collection scheme TM^2^ was proposed first in Wu et al. [2017b] and later expanded by Wu et al. [2017a]. We describe our modified basic version of the TM^2^ method here:

Step 1. The data collectors plan the data collection, create the database structure, program the data collection system. They randomly generate a *p* × *p* random orthogonal matrix ***B***, which is distributed to the participants’ data collection devices.

Step 2. Each participant’s data *x*_1_ (a vector of dimension *p*_1_) is collected and merged with Gaussian noise *x*_2_ (of dimension *p*_2_) into a vector *x* = (*x*_1_*, x*_2_) of dimension *p* = *p*_1_ + *p*_2_. Then *x* is right multiplied by ***B*** on the participant’s device, and only the resulting masked data *x****B*** leaves the device and is sent to the masking service provider.

Step 3. The masking service provider generates another *n* × *n* random orthogonal matrix ***A***_2_. After receiving data from all participants, it combines the individual data *x****B*** into a *n* × *p* matrix ***XB***, left multiplies by ***A***_2_ and sends the doubly masked data ***A***_2_***XB*** to the data collectors.

Step 4. The data collectors multiply ***A***_2_***XB*** by ***B***^−1^ to get back ***A***_2_***X*** and take the first *p*_1_ columns to get ***A***_2_***X***_1_. Then the data collectors generate another *n* × *n* random orthogonal matrix ***A***_1_, left multiply it to ***A***_2_***X***_1_, and publish ***AX***_1_ (where ***A*** = ***A***_1_***A***_2_) which is accessible by all data users.

Detailed theoretical analysis of the privacy guarantee on the TM^2^ method has been missing. This paper fills that gap by proving theoretically that this modified version of the TM^2^ method is strong obfuscating. We prove the strong obfuscating guarantee by showing that: (A) the extra information that any party in the process owns will not allow the party to reduce the data domain (possible values of data) small enough to identify individual level data; and (B) there is no statistical information leakage beyond the domain restriction considered in (A).

Compared to the original TM^2^ scheme in Wu et al. [2017b], we make two modifications on the TM^2^ procedure. For the first modification, we add random Gaussian noise in Step 2. The data collector wants to collect *p*_1_ variables on *n* individuals so that the real response matrix becomes ***X***_1_ of dimensions *n* × *p*_1_. We ask each participant to generate *p*_2_ pure Gaussian noise variables, on his/her device according to a fixed variance parameter *σ*^2^. Hence, the full data matrix would be ***X*** = (***X***_1_, ***X***_2_). For privacy protections proved in later sections, we require that *p*_1_ < *n* ≤ *p* = *p*_1_ + *p*_2_. In Step 2 of this modified procedure, Gaussian noise *x*_2_ is mingled with real response *x*_1_ to provide protection in addition to the random mask ***B***. In Step 4, after the collectors get back ***A***_2_***X*** = (***A***_2_***X***_1_, ***A***_2_***X***_2_), they separate the matrix and discard those noises. Therefore the published data set ***AX***_1_ with ***A*** = ***A***_1_***A***_2_ still gives the exact summary statistic, as it is only masked by ***A*** without containing the added noise.

For the second modification, instead of using a random invertible matrix for the right mask ***B*** as originally proposed by Wu et al. [2017b], we use a random orthogonal matrix for the right mask ***B***. As we will see in the privacy analysis in the next section, using an invertible matrix does make one part of the privacy proof easier. However, the other part of privacy proof depends on using a uniformly distributed random matrix to avoid information leakage that can lead to probabilistic attacks. While there is a natural uniform distribution on all orthogonal matrices that is well studied in literature, there is no natural uniform distribution on the set of all invertible matrices. The uniformly distributed orthogonal matrix ***B*** does provide sufficient privacy protection when combined with the addition of noise ***X***_2_.

### 2.1. Privacy Analysis of TM^2^etup.

To rigorously study the privacy protection issues in this data collection process, we analyze the information that can be accessed by each party and analyze whether such information allows inference of the individual level data.

First, we illustrate how to analyze the privacy protection assuming that the adversary only has access to the publicly published left-masked data set ***M****_L_* = ***AX***_1_ where ***A*** is a random *n* × *n* orthogonal matrix. The issue becomes whether an adversary can identify individual level data knowing only ***M****_L_* = ***m****_L_*.

We consider the analysis in two stages. When given ***M****_L_* = ***m****_L_*, this restricts the possible values of ***X***_1_ and can thus reveal information. In the first stage, we consider whether this support restriction on ***X***_1_ (due to ***M****_L_* = ***m****_L_*) enables the identification of individual data. Let $S_{X_{1}}$ denote the support of ***X***_1_, and let $S_{X_{1}}\left(m_{L}\right)$ denote the restricted support of ***X***_1_ given that ***M****_L_* = ***m****_L_*. The privacy preservation depends on the size of $S_{X_{1}}\left(m_{L}\right)$. For example, in an extreme case, if the $S_{X_{1}}\left(m_{L}\right)$ contains only one matrix, then ***X***_1_ is known to everyone and data privacy cannot be protected. Generally we can show, in next section, that this restricted support $S_{X_{1}}\left(m_{L}\right)$ is big enough so that identification of individual data is impossible.

In the second stage, we consider whether the adversary can learn any information beyond the restriction on support which was analyzed in the first stage. Such information can enable adversaries to launch probabilistic attacks [Machanavajjhala et al., 2007, Fung et al., 2010]. Fortunately, due to the independence between the mask ***A*** and the raw data ***X***_1_, we can show that the posterior density of ***X***_1_ given ***M****_L_* = ***m****_L_* is the same as the prior density of ***X***_1_ restricted to the support $S_{X_{1}}\left(m_{L}\right)$. Thus any loss of privacy is through the support restriction already studied in stage one. Therefore, knowing ***M****_L_* = ***m****_L_* does not identify individual level data.

Next, we consider the privacy protection for all parties involved in the whole TM^2^ data collection process. That is, we conduct the above two-stage privacy protection analysis given all information available to one party in the process. The data collector and the masking service provider each have access to some intermediate masked data in addition to the public data. Hence, we need to analyze privacy protection for an adversary knowing this intermediate masked data together with the public data set ***M****_L_* = ***AX***_1_.

The data collector knows, in addition to ***M****_L_* = ***AX***_1_, the double masked data ***A***_2_***XB***. Since the data collector knows the masks ***A***_1_ and ***B***, knowing these data ***A***_1_***A***_2_***X***_1_ and ***A***_2_***XB*** are simply equivalent to knowing ***A***_2_***X***. Due to the fact that ***X***_2_ is purely noise independent of raw data ***X***_1_, the theoretical privacy analysis for the data collector knowing ***A***_2_***X*** = (***A***_2_***X***_1_, ***A***_2_***X***_2_) will have basically the same results as the analysis for the user with access only to ***M****_L_* = ***AX***_1_.

The masking service provider has access to the right-masked data ***M****_R_* = ***XB*** in addition to the public left-masked data ***M****_L_* = ***AX***_1_. This information results in the most severe restriction on the support when compared to what resulted from knowledge by other parties. Thus, this is the weakest link for privacy preservation in the whole TM^2^ data collection scheme. In Section 3, we present details of the two-stage privacy protection analysis when both ***M****_L_* and ***M****_R_* are known.

## 3. Theoretical Analysis of Privacy Preservation of TM^2^

### 3.1. Notations, Formalizations and Technical Preliminaries.

We denote the probability densities of random matrices ***X***_1_, ***X***_2_, ***A*** and ***B*** as $\pi_{X_{1}}\left(x_{1}\right)$, $\pi_{X_{2}}\left(x_{2}\right)$, *π****_A_***(***a***) and *π****_B_***(***b***) respectively. The supports of these distributions are denoted respectively as $S_{X_{1}}$, $S_{X_{2}}$, $S_{A}$ and $S_{B}$.

We want to study, based on information *INFO* available to one party, what this party can infer about the individual level data. Here this *INFO* includes the publicly available final left-masked data ***M****_L_* = ***AX***_1_ and some extra information available to the particular party. The restricted support of ***X***_1_ given *INFO* is denoted as $S_{X_{1}}\left(INFO\right)$ which consists of all *n* × *p* matrices that can be the value of ***X***_1_ which is compatible with *INFO*.

For example, given only the public masked data *INFO* = ***M****_L_*, the restricted support is

$$S_{X_{1}}\left(M_{L}\right)=\left\{U:\exists \tilde{A}\in S_{A}\;\text{such}\;\text{that}\;\tilde{A}U=M_{L}.\right\}$$

Let $O_{n}$ denote the set consisting of all orthogonal matrices. In the case of left masking with a random orthogonal matrix ***A***, for any orthogonal $\bar{A}\in O_{n}$, $U=\bar{A}X_{1}$ is compatible with *INFO* = ***M****_L_*. That is, $S_{X_{1}}\left(M_{L}\right)=O_{n}X_{1}$. To see this, let $\tilde{A}=A{\bar{A}}^{T}$, then $\tilde{A}\in O_{n}$ and $\tilde{A}\left(\bar{A}X_{1}\right)=M_{L}$. Here and throughout this paper, we use *^T^* to denote the transpose of a matrix.

For the strong obfuscating guarantee, we wish to show that the extra information available to the parties in the process does not cause any privacy loss more than the publicly released final left-masked data ***M****_L_* = ***AX***_1_. We want to show that: stage one (i) the restricted support $S_{X_{1}}\left(INFO\right)$ is the same as $S_{X_{1}}\left(M_{L}\right)=O_{n}X_{1}$; stage two (ii) the conditional probability distribution of ***X***_1_ given *INFO* is similar to the probability distribution of ***X***_1_ given support $S_{X_{1}}\left(INFO\right)$, thus there is no privacy loss through probability attacks beyond the loss from the support restriction considered in stage one.

We now formalize the precise mathematical statements to prove in stages one and two. More precisely for stage one, we hope that the restricted support is the same as if only the public left-masked data is available.

(i) $$S_{X_{1}}(INFO)=O_{n}X_{1},$$

for *INFO* available to any one party in the process. For the second stage, we denote $\pi_{X_{1}|INFO}\left(x_{1}|INFO\right)$ as the posterior distribution of ***X***_1_ given *INFO*. The prior density $\pi_{X_{1}}$ restricted on the support $S_{X_{1}}\left(INFO\right)$ is

$$\pi_{X_{1}|S}{{}_{{}_{X_{1}}}}_{\left(INFO\right)}\left(x_{1}\right)=\frac{\pi_{X_{1}}\left(x_{1}\right)}{\int_{S_{X_{1}}(INFO)}\pi_{X_{1}}\left(x_{1}^{*}\right)dx_{1}^{*}}.$$

To show that there is no extra privacy loss beyond the support restriction considered in stage one, we prove that these two probability densities agree with each other. That is, we wish to prove

(ii) $$\pi_{X_{1}|INFO}\left(x_{1}|INFO\right)=\pi_{X_{1}|S}{{}_{{}_{X_{1}}}}_{\left(INFO\right)}\left(x_{1}\right).$$

#### Definition 3.1.

A data collection process is *strong collection obfuscating* if conditions (*i*) and (*ii*) hold for the information *INFO* available to any party in this process.

A slightly weaker version is that the above property holds with a high probability. Notice that the *INFO* available to any party in this process can be determined from the values of ***X***_1_, ***X***_2_, ***A*** and ***B*** which are generated respectively from distributions with densities $\pi_{X_{1}}\left(x_{1}\right)$, $\pi_{X_{2}}\left(x_{2}\right)$, *π****_A_***(***a***) and *π****_B_***(***b***). Thus such *INFO* is generated from a probability distribution defined by $\pi_{X_{1}}\left(x_{1}\right)$, $\pi_{X_{2}}\left(x_{2}\right)$, *π****_A_***(***a***) and *π****_B_***(***b***). We want that, with high probability from this probability distribution, the generated values of *INFO* satisfy conditions (*i*) and (*ii*).

#### Definition 3.2.

A data collection process is *ϵ-strong collection obfuscating* if, with probability at least 1 − *ϵ*, conditions (*i*) and (*ii*) hold for the information *INFO* available to any party in this process.

Our definition of the strong collection obfuscating procedure ensures that there is no privacy loss due to observations by any party in the process beyond those contained in the publicly released final data. This definition delineates the privacy protection in the collection process from the privacy protection in the publicly releasing of final data ***M****_L_* = ***AX***_1_. The theoretical analysis concentrates on the soundness of the collection process.

Given the public left-masked data ***M****_L_* = ***AX***_1_, the statistics $X_{1}^{T}X_{1}$ are released to the user. The user has the first two exact statistical moments and statistical models, such as linear regression, can be fitted exactly as if the user has the raw data set ***X***_1_. And the residuals are known up to an orthogonal matrix multiplication, therefore the usual statistical model diagnostics methods can also be carried out as if done on the raw data set.

For continuous data, the user cannot recover the individual level data since the user only sees a linear combination of all individuals’ data, and there is no utilizable statistical distributional information other than the prior (population) density $\pi_{X_{1}}$. This ensures the privacy of individual data.

In practice, the types of elements in ***X***_1_ may also be known to the user. This can further restrict the support. We assume that the elements in data matrix ***X***_1_ are all encoded as numerical values (e.g., “yes/no” answer to a question may be encoded as 1 and 0). We consider that the type of data in each column, either as continuous/discrete/binary, is public knowledge. Let $S_{j}$ denote the support of the type of data in the *j*-th column of ***X***_1_. For example, if data are continuous, then $S_{j}=R$; if the data are binary, then $S_{j}=\{0,\;1\}$; if the data are positive integers, then $S_{j}=\{1,2,\ldots \}=N^{+}$. Knowing the type of data in each column would restrict the support of ***X***_1_ to

$${\tilde{S}}_{X_{1}}=\{U:\;\text{(all}\;\text{entries}\;\text{of}\;j\text{-th}\;\text{column}\;\text{in}\;U)\in S_{j}\;j=1,\ldots ,p_{1}.\}.$$

Then with knowledge of both *INFO* and types of data, the restricted support becomes the intersection of ${\tilde{S}}_{X_{1}}$ and $S_{X_{1}}\left(INFO\right)$,

$$S_{X_{1}}(INFO;\;TYPE)={\tilde{S}}_{X_{1}}\cap S_{X_{1}}(INFO).$$

Let $P_{n}$ denote the set of all permutation matrix ***P***. Since all permutation matrices are orthogonal and permutation does not change the type of elements, we have the following Lemma:

#### Lemma 3.3.

For any strong obfuscating data collection process,

$$P_{n}X_{1}\subseteq \left[{\tilde{S}}_{X_{1}}\cap O_{n}X_{1}\right]=S_{X_{1}}(INFO;\;TYPE).$$

Lemma 3.3 indicates that a strong collection obfuscating data collection process offers some privacy protection even when the data types are known. Since all permutations are in the $S_{X_{1}}(INFO;\;TYPE)$, any individual cannot be identified here without extra side information. It is not clear whether the type can be combined with some side information (such as data that a particular individual is a smoker) to reveal other individual level data. However, notice that any weakness in this aspect is inherently due to releasing the public data ***AX***_1_. Our strong collection obfuscating procedure ensures that no extra privacy loss is added during the process beyond the privacy loss in releasing ***AX***_1_.

As we discussed in the previous section, the party with the most information during the *TM*^2^ process is the masking service provider who knows *INFO* = (***M****_L_,* ***M****_R_*). Therefore, in the next section, we study when (*i*) and (*ii*) hold for *INFO* = (***M****_L_,* ***M****_R_*). Here we first state some technical preliminary results on the characterization of the uniform distribution on orthogonal matrices. These preliminary results are used in studying the second stage condition (ii) later.

Under the matrix multiplication, the orthogonal matrices form a compact Hausdorff topological group $O_{n}$. Therefore, there is a unique Haar measure *μ*(·) on $O_{n}$ such that the measure of the whole sample space $O_{n}$ equals one. Then this Haar measure induces a natural uniform distribution on $O_{n}$. See Chapter 2 of Zhang [2014] for a detailed technical equivalent characterization of the uniform distribution on $O_{n}$. Since a Haar measure *μ*(·) is invariant under the matrix multiplication, the uniform distribution is also invariant under the matrix multiplication.

#### Lemma 3.4.

*Let π*_0_(·) *denote the probability density function of the uniform distribution on* $O_{n}$. *Then for any orthogonal matrix* $A_{0}\in O_{n}$,

(3.1) $$\pi_{0}(a)=\pi_{0}\left(A_{0}a\right)=\pi_{0}\left(aA_{0}\right),\;\;for\;all\;a\in O_{n}.$$

Also, the product of two uniformly distributed orthogonal matrices is also uniformly distributed.

#### Lemma 3.5.

*If* ***A***_1_ ~ *π*_0_ *and* ***A***_2_ ~ *π*_0_ *are independent of each other, then their product* ***A*** = ***A***_1_***A***_2_ *also follows the uniform distribution π*_0_ *on* $O_{n}$.

The proof is straightforward and can be found in Chapter 2 of Zhang [2014].

In the TM^2^ scheme, when the masking service provider and the data collector each generate a random orthogonal matrix ***A***_1_ and ***A***_2_ respectively according to *π*_0_, then the mask ***A*** = ***A***_1_***A***_2_ for the publicly released data set is also uniformly distributed. In practice, the uniformly distributed random orthogonal matrices can be generated using algorithms described in Heiberger [1978], Anderson et al. [1987], Wu et al. [2017b].

### 3.2. Restricted Support Given Knowledge of Masked Data Sets.

We first prove that condition (i) holds for *INFO* = (***M****_L_,* ***M****_R_*) when invertible matrices are used for right mask ***B*** as originally proposed by Wu et al. [2017b]. That is, $B\in I_{n}$, where $I_{n}$ denote the set of all *n* × *n* invertible matrices. Condition (i) then becomes that all orthogonal transformations of ***X***_1_ are contained in the restricted support

(3.2) $$S_{X_{1}}\left(M_{L},M_{R}\right)=\{U:\exists \tilde{A}\in S_{A},\tilde{B}\in S_{B}\;\text{and}\;\tilde{U}\in S_{X_{2}}\;\text{such}\;\text{that}\;\;\;\;\;\;\;\;\;\;\;\;\;\;\;\;\;\;\;\;\;\;\;\;\;\;\;\;\;\;\;\;\;\;\;\;\;\;\;\;\;\;\;\;\;\;\;\;\;\;\;\;\tilde{A}U=M_{L}\;\text{and}\;(U,\tilde{U})\tilde{B}=M_{R}\}.$$

#### Theorem 3.6.

*Suppose* $S_{A}=O_{n}$ *and* $S_{B}=I_{p}$, *p*_1_ ≤ *n* ≤ *p and* ***X*** *is full rank (i.e., rank*(***X***) = *n), then for any* $P\in O_{n}$, $PX_{1}\in S_{X_{1}}\left(M_{L},\;M_{R}\right)$. *In other words,* $S_{X_{1}}\left(M_{L},\;M_{R}\right)=O_{n}X_{1}=O_{n}M_{L}$.

##### Proof.

We need to show that, for any $P\in O_{n}$, $U\;=\;PX_{1}\in S_{X_{1}}\left(M_{L},\;M_{R}\right)$.

Since $P\in O_{n}$ and $A\in O_{n}$, then $\tilde{A}=AP^{T}\in O_{n}$. Then we have

(3.3) $$\tilde{A}U=AP^{T}PX_{1}=AX_{1}=M_{L}.$$

Since ***X*** is full-rank and *n* ≤ *p*, there exists a (*p* − *n*) × *p* matrix ***X***∗ such that $\left(\begin{array}{c} X\\ X* \end{array}\right)$ is full-rank and thus invertible. Since $P\in O_{n}$, $\left(\begin{array}{c} P^{T}X\\ X* \end{array}\right)$ is also full-rank and invertible. Hence we can define an invertible matrix

$$\tilde{B}={\left(\begin{array}{c} X\\ X* \end{array}\right)}^{-1}\left(\begin{array}{c} P^{T}X\\ X* \end{array}\right)B.$$

Also let $\tilde{U}=PX_{2}$ thus $(U,\;\tilde{U})=PX$, we have

$$\left(\begin{array}{c} \left(U,\;\tilde{U}\right)\\ X* \end{array}\right)\tilde{B}=\left(\begin{array}{cc} P & 0\\ 0 & I \end{array}\right)\left(\begin{array}{c} X\\ X* \end{array}\right)\tilde{B}=\left(\begin{array}{cc} P & 0\\ 0 & I \end{array}\right)\left(\begin{array}{c} P^{T}X\\ X* \end{array}\right)B=\left(\begin{array}{c} X\\ X* \end{array}\right)B,$$

where ***I*** is the identity matrix of size (*p* − *n*) × (*p* − *n*). The first *p* rows in the last equation are

(3.4) $$(U,\tilde{U})\tilde{B}=XB=M_{R}.$$

(3.3) and (3.4) together means that ***U*** satisfies (3.2), thus ***U*** belongs to $S_{X_{1}}\left(M_{L},\;M_{R}\right)$. □

Theorem 3.6 states that condition (i) is satisfied when the ***X*** is full rank. In the original TM^2^ scheme proposal, the full rank condition may or may not be satisfied because it is determined by the underlying probability distribution of ***X***_1_ which is outside the control of the designer of this procedure. With the modification of extra noise matrix ***X***_2_, we can ensure the full rank condition by specifying the noise generation mechanism. Particularly, we specify that each individual data provider generates a *p*_2_-dimension noise vector with i.i.d. elements from a Gaussian distribution with *p*_2_ ≥ *n*. This will ensure with probability one that ***X*** is indeed full rank.

#### Remark 1. (Size of the right mask)

*For privacy preservation, the size of right mask p has to be bigger than the data size n as assumed in Theorem 3.6. When p* < *n, some rows of* ***M****_R_ are linear dependent which provides further restriction on the support. We provide such a counter example in Appendix A to illustrate that such a restriction together with knowledge of data type can reveal individual level data.*

Above we considered the support restriction under the original TM^2^ scheme proposal [Wu et al., 2017b] of invertible right mask, $S_{B}=I_{p}$. However, unlike $O_{p}$, $I_{p}$ does not form a compact Hausdorff topological group. Therefore, there exists no uniform distribution on $I_{p}$. Due to the non-uniformity of ***B***, the posterior distribution of ***X***_1_ given (***M****_L_,* ***M****_R_*) leaks information beyond the support restriction, thus the second stage condition (*ii*) no longer holds. This makes the usage of random invertible right masks in the TM^2^ scheme very tricky. It is unclear what distribution on $I_{p}$ should be used to generate the random invertible ***B***.

Here, we consider the modification of the TM^2^ scheme where the right mask ***B*** is a random orthogonal matrix generated from the uniform distribution *π*_0_ on $O_{p}$. We show that if the random noise ***X***_2_ is large enough, then condition (*i*) still holds when the orthogonal right mask ***B*** is used.

Let *λ_min_*(***M***) and *λ_max_*(***M***) denotes the minimum and the maximum eigenvalues of a semi-positive definite matrix ***M***. The restricted support will remain big if the noise is large enough:

(3.5) $$\lambda_{min}\left(M_{R}M_{R}^{T}-X_{1}X_{1}^{T}\right)=\lambda_{min}\left(X_{2}X_{2}^{T}\right)>\lambda_{max}\left(X_{1}X_{1}^{T}\right).$$

Now we have a result similar to Theorem 3.6.

#### Theorem 3.7.

*Suppose* $S_{A}=O_{n}$ *and* $S_{B}=O_{p}$, *p*_1_ ≤ *n* ≤ *p. If condition* (3.5) *holds, then* $S_{X_{1}}\left(M_{L},\;M_{R}\right)=O_{n}X_{1}$.

The proof is provided in Appendix B.

Next, we show that condition (*ii*) also holds when under condition (3.5). Then we discuss how achievable the technical condition (3.5) is in practice.

### 3.3. Information Leakage Beyond the Support Restriction.

We now study the second stage condition (*ii*) by checking the amount of information an adversary can get from the posterior distribution of ***X***_1_ given (***M****_L_,* ***M****_R_*) beyond their restriction on the support of ***X***_1_. Given *INFO* = (***M****_L_,* ***M****_R_*), the posterior density is denoted as $\pi_{X_{1}|\left(M_{L},M_{R}\right)}\left(x_{1}|m_{L},m_{R}\right)$. The prior density $\pi_{X_{1}}$ restricted on the support $S_{X_{1}}\left(m_{L},\;m_{R}\right)$ is denoted as $\pi_{X_{1}|S}{}_{{}_{X_{1}}}\left(m_{L},m_{R}\right)$.

#### Theorem 3.8.

*Let* ***X***_1_ *be a random matrix with probability density $\pi_{X_{1}}$. We assume that the elements in* ***X***_2_ *are generated i.i.d. from a Gaussian distribution with mean zero. When condition* (3.5) *holds, given* ***M****_L_ and* ***M****_R_, the posterior density of* ***X***_1_ *is the same as the prior density restricted on $S_{X_{1}}\left(M_{L},\;M_{R}\right)$. That is,*

(3.6) $$\pi_{X_{1}|\left(M_{L},M_{R}\right)}\left(x_{1}|m_{L},m_{R}\right)=\pi_{X_{1}|S}{}_{{}_{X_{1}}\left(m_{L},m_{R}\right)}\left(x_{1}\right).$$

The proof of Theorem 3.8 is provided in Appendix D.

### 3.4. *ϵ*-strong obfuscating TM^2^.

Theorem 3.7 and Theorem 3.8 states, respectively, that conditions (*i*) and (*ii*) hold under condition (3.5). Combining them, we have the following Theorem.

#### Theorem 3.9.

*If*

(3.7) $$Pr\left[\lambda_{min}\left(X_{2}X_{2}^{T}\right)>\lambda_{max}\left(X_{1}X_{1}^{T}\right)\right]\geq 1-\epsilon ,\;$$

*then the proposed TM*^2^ *procedure is ϵ-strong collection obfuscating by* Definition 3.2.

The *ϵ*-strong collection privacy property ensures that there is at most *ϵ* probability for the process to leak any privacy information beyond the public released data ***AX***_1_. TM^2^ achieves this property when the technical condition (3.7) holds. To achieve the technical condition (3.7), we generate the *p*_2_-dimensional noise vector *x*_2_ with i.i.d. Gaussian elements of mean zero and a sufficiently large variance *σ*^2^. We present a technical probability bound in Appendix C, where the probability of violating condition (3.5) is decreasing exponentially and specifics a *σ*^2^ value which ensures condition (3.7). Larger variance *σ*^2^ always increases the probability that condition (3.5) holds. In practice, the variance *σ*^2^ is only limited due to the computation accuracy. That is, *σ* should not exceed raw data values by the orders of magnitude allowed by the machine precision.

### 3.5. Extension to Alleviate Collusion Risks.

We have shown that the privacy of individual data can be protected when no party in the TM^2^ scheme knows all the masks. However, there are also risks of collusion among different parties in the procedure. Since the right mask ***B*** is known to the data collector and all individual data providers, if one of them share this info with the masking services provider, then the privacy protection can be broken.

Wu et al. [2017a] proposed ideas to protect against this collusion risk using the ideas of multiparty computation. For each individual, the data vector *x* can be broken up as *K*_1_ random components $x^{1},\ldots ,x^{K_{1}}$ where $x=x^{1}+\ldots +x^{K_{1}}$. Then such components are sent to *K*_1_ right masking service providers, one to each. The resulting masked data *x^i^****B****_i_*, *i* = 1, ..., *K*_1_, are then sent to the left masking service provider to be merged and created the double masked data ***AX****^i^****B****_i_*, *i* = 1, ..., *K*_1_. For further protection, they can be passed through *K*_2_ left masking service providers to generate $A_{K_{2}}\ldots A_{1}X^{i}B_{i}$. Let $A=A_{K_{2}}A_{K_{2}-1}\ldots A_{1}$. Then the masked data ***AX****^i^****B****_i_*, *i* = 1, ..., *K*_1_, are sent to the corresponding right masking service providers to remove the right masking. Then the resulting ***AX****^i^*, *i* = 1, ..., *K*_1_, are sent to the data collector to generate $AX=AX^{1}+\ldots +AX^{K_{1}}$. Unless all *K*_1_ right (or all *K*_2_ left) masking service providers collude, they cannot find values of all components $X^{1},\ldots ,X^{K_{1}}$.

The stage one theoretical analysis on this extended TM^2^ scheme can be analyzed similarly as before, where the restricted support condition (i*) holds given condition (3.5). The stage two analysis is more involved, as the posterior distribution of ***X***_1_ given some shares, depends on the distribution of the shares. Which random distributions should the shares be generated from to effect no additional privacy loss remains an open question, and will be investigated in future work.

## 4. Discussions and Conclusions

This paper conducts a theoretical analysis of privacy preservation in a modified TM^2^ scheme. Random noises were used with uniformly distributed orthogonal matrix masks to hide individual data during the data collection process. The noise addition in the first step of the TM^2^ scheme is similar to the idea of noise perturbed response schemes. However, the critical difference is that our noise addition is only intended to help mask data during the transition, and is in fact removed after the right mask removal. The resulting published data set is a left masked data set with exact summary statistics, unlike many other noise addition schemes where the summary statistics are randomly approximated.

This work is aimed to protect against unscrupulous access to the raw data ***X***_1_ traditionally hold by a trusted operator. We would like to further clarify the relationship to differential privacy methods [Dwork et al., 2006] which aims to provide a strong privacy protection and closure under composition of multiple accesses to the database. There are two types of differential privacy models. In the central model, a trusted database operator holds the raw data, and releases noise perturbed summary statistics for inquires. In the local model [Evfimievski et al., 2003, Dwork et al., 2006, Kasiviswanathan et al., 2011, Cormode et al., 2018], noise is added at the individual level based on the idea of randomized response methods [Warner, 1965, Blair et al., 2015]. The local differential privacy procedures similarly addresses the issue of untrustworthy central database operator. In recent years, Goolge [Erlingsson et al., 2014], Apple [Thakurta et al., 2017] and Microsoft [Ding et al., 2017] have all developed and deployed local differential privacy procedures in data collection.

There are two type of possible unscrupulous access to the raw data ***X***_1_ to be addressed. The first is that the data collector is untrustworthy. The second is that an unscrupulous party might break in to the server containing data collected by an honest data collector. In the differential privacy literature, the first type is handled by using local differential privacy procedures, while the second type is addressed via pan-private data analysis [Dwork et al., 2010]. Our TM^2^ scheme protects against both type of unscrupulous accesses, but only allow for a one-shot collection for each individual’s data.

While both the local differential privacy procedures and the TM^2^ scheme can provide protection against unscrupulous accesses, the goals are somewhat different. The TM^2^ scheme aims to collect a masked data set that preserves the first two statistical moments of the variables (note that $X_{1}^{T}X_{1}$ is knowable from the publicly available ***AX***_1_). This allows exact statistical inferences on quantities depending on these statistical moments. The local differential privacy methods, on the other hand, aims to provide a stronger privacy protection under composition of multiple data collections/accesses.

The idea of the TM^2^ scheme is similar to secure multi-party computation (SMC) procedures, in that this scheme tries to distribute information among parties so that each party does not get access to individual level data other than its own. There are also important differences between TM^2^ and SMC. They differ in their designed purposes even though both want parties to cooperate in a joint task while keeping privacy. SMC is designed to conduct joint statistical analysis without the parties revealing their data to each other. TM^2^ wants to collect the masked data set, which enables statistical analysis, without parties revealing the actual data to the data collector. Operationally, SMC requires distributed storage of data as well as distributed computation. Specifically, if we require that the private data of parties never leave their devices, then SMC needs the parties to stand by ready for any statistical analysis that may occur much later in the future. In contrast, the TM^2^ method is only distributive in the data collection stage. The private data leaves the parties’ devices in a masked form, and later is centrally stored in masked form ***AX***_1_. Since all future statistical analysis is conducted on the publicly released ***AX***_1_, there is no need for the parties to be available for future analysis.

In this paper, we presented a privacy analysis clearly separating the risks coming from support restriction and the risks of probabilistic attacks beyond the support restriction. With the analysis, we show that the TM^2^ scheme is safe to collect a synthetic data set ***AX***_1_ which is a random orthogonal transformation of the raw data set ***X***_1_. All information during the data collection procedure is masked, and no one during the procedure can access the raw data set. This removes the issue of trusting a data record keeper and provides a new tool for researchers to collect data allowing exact statistical inference for linear models while provide a privacy protection: no hacking attack against a party in the data collection procedure can access real individual level data since all parties do not have enough information to infer the private individual data.

## Figures and Tables

**Figure 1:**
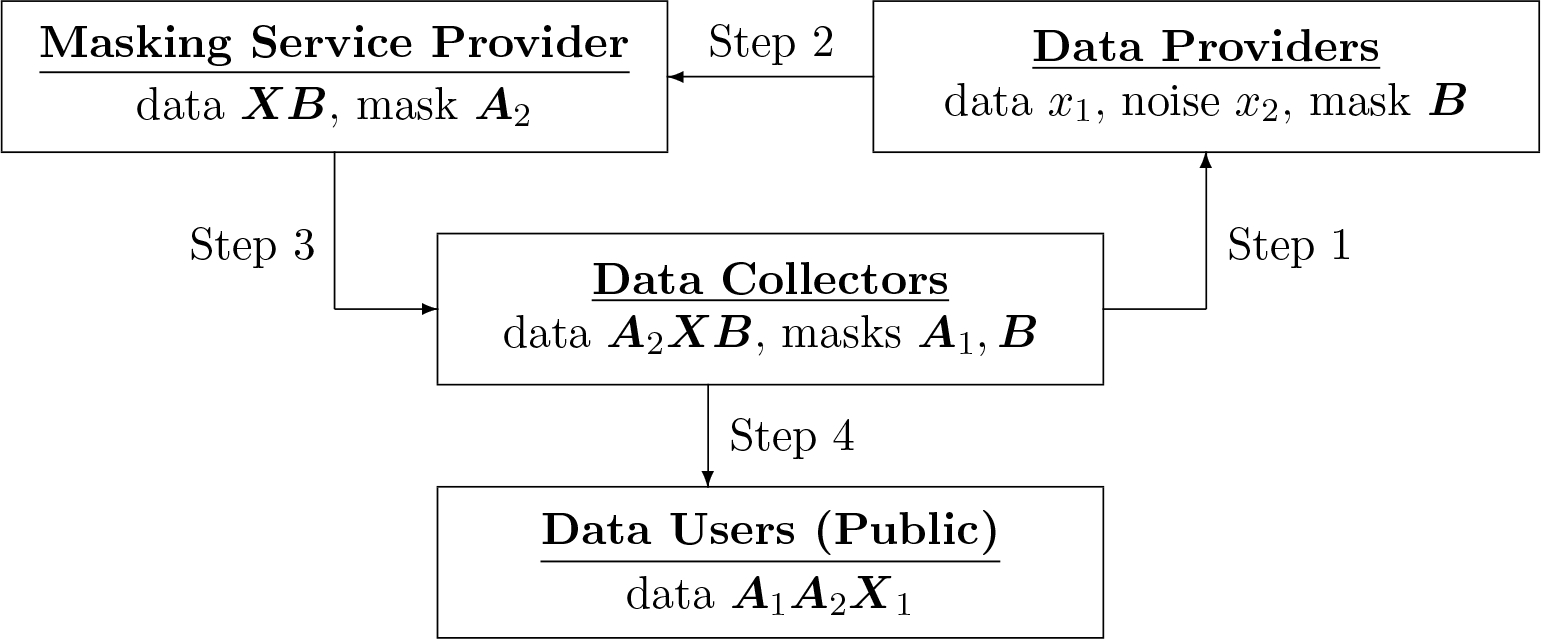
*This diagram shows each party’s knowledge about the data and the masking matrices in the modified TM*^2^ *method. Each party knows some masked version of the data:* ***XB*** *for the masking service provider,* ***A***_2_*X for the data collector, and* ***A***_1_***A***_2_***X***_1_ *for everybody including the public. Nobody knows the original data* ***X***_1_*, with each data provider (participant) knowing only his/her row x*_1_

## Appendix A. A Counter Example

We illustrate that *p* ≥ *n* and the full rank condition on ***X*** are needed for the privacy preservation in the TM^2^ scheme through a simple counter example here. We consider a 3 × 2 matrix ***X***, where the first column ***X***_1_ contains binary sensitive information and the second column ***X***_2_ contains continuous random noise. Suppose that only one of the three individuals answered “1” on the sensitive question, so that the data matrix is

(A.1) $$X=\left(\begin{array}{ll} x_{11} & x_{12}\\ x_{21} & x_{22}\\ x_{31} & x_{32} \end{array}\right)=\left(\begin{array}{ll} 1 & a_{1}\\ 0 & a_{2}\\ 0 & a_{3} \end{array}\right).$$

We decompose $X=\left(\begin{array}{l} X_{a}\\ X_{b} \end{array}\right)$ with the first two rows as ***X****_a_* and the last row as ***X****_b_*. Without loss of generality, we assume that *a*_2_ ≠ 0 so that $\left(\begin{array}{ll} 1 & a_{1}\\ 0 & a_{2} \end{array}\right)$ is non-singular, and we assume that *a*_3_*/a*_2_ is not an integer. Then the first column ***X***_1_ = (1, 0, 0)*^T^* can be uniquely determined from the masked data ***M****_R_*.

To see this, we decompose $M_{R}=\left(\begin{array}{l} M_{a}\\ M_{b} \end{array}\right)$ similarly as in the decomposition of $X\;=\;\left(\begin{array}{l} X_{a}\\ X_{b} \end{array}\right)$. Then $M_{b}M_{a}^{-1}=\left(X_{b}B\right){\left(X_{a}B\right)}^{-1}=X_{b}BB^{-1}X_{a}^{-1}=X_{b}X_{a}^{-1}$ is known to anyone with access to ***M****_R_*. Using (A.1), this means $X_{b}X_{a}^{-1}=\left(0,\;a_{3}/a_{2}\right)$ is determined from the masked data ***M****_R_*. Then the first element in $\left(X_{b}X_{a}^{-1}\right)X_{a}=X_{b}$ indicates that (0*, a*_3_*/a*_2_)(*x*_11_*, x*_21_)*^T^* = *x*_31_. That is, (*a*_3_*/a*_2_)*x*_21_ = *x*_31_.

Since *x*_21_ and *x*_31_ are binary entries in ***X***_1_ and *a*_3_*/a*_2_ is not an integer, the attacker can infer from (*a*_3_*/a*_2_)*x*_21_ = *x*_31_ that *x*_21_ = *x*_31_ = 0. Then we must have *x*_11_ = 1 due to ***M****_R_* (and thus ***X***) being full-rank. That is, we now know every entry in ***X***_1_ = (1, 0, 0)*^T^* from the masked data ***M****_R_*.

Notice that according to Lemma 3.3, a strong collection obfuscating procedure would not have allowed this identification of individual data from the random permutation. There is indeed additional privacy loss without assuming *p* < *n*. In general, when *p* < *n* and ***M****_R_* is full rank, $\left(M_{b}M_{a}^{-1}\right)X_{a}=X_{b}$ along with knowledge of the data type may leak sensitive information about original data ***X***.

## Appendix B. Proof of Theorem 3.7.

### Proof.

Same arguments in proof of Theorem 3.6 shows that (3.3) holds. Therefore we only need to show that there exist ${\tilde{X}}_{2}$ and $\tilde{B}$ satisfying (3.4): $\left(PX_{1},\;\tilde{U}\right)\tilde{B}=XB=M_{R}$.

Using condition (3.5), we have

$$\lambda_{min}\left(X_{1}X_{1}^{T}+X_{2}X_{2}^{T}-PX_{1}X_{1}^{T}P^{T}\right)\geq \lambda_{min}\left(X_{1}X_{1}^{T}\right)+\lambda_{min}\left(X_{2}X_{2}^{T}\right)-\lambda_{max}\left(PX_{1}X_{1}^{T}P^{T}\right)\;\;\;\;\;\;\;\;\;\;\;\;\;\;\;\;\;\;\;\;\;\;\;\;\;\;\;\;\;\;\;\;\;\;\;\;\;\;\;\;\;\;\;\;\;\;\;\;\;\;\;\;\;\;\;\;\;\;\;\;\;\;\;\;\;\;\;\;\;\;\;\;\;\;\;\geq \lambda_{min}\left(X_{2}X_{2}^{T}\right)-\lambda_{max}\left(X_{1}X_{1}^{T}\right)\;\;\;\;\;\;\;\;\;\;\;\;\;\;\;\;\;\;\;\;\;\;\;\;\;\;\;\;\;\;\;\;\;\;\;\;\;\;\;\;\;\;\;\;\;\;\;\;\;\;\;\;\;\;\;\;\;\;\;\;\;\;\;\;\;\;\;\;\;\;\;\;\;\;\;\text{>0}.$$

Hence $X_{1}X_{1}^{T}+X_{2}X_{2}^{T}-PX_{1}X_{1}^{T}P^{T}$ is a positive definite matrix. Therefore, there exists a matrix $\tilde{U}$ such that

(B.1) $$\tilde{U}{\tilde{U}}^{T}=X_{1}X_{1}^{T}+X_{2}X_{2}^{T}-PX_{1}X_{1}^{T}P^{T}.$$

This is equivalent to

(B.2) $$\left(PX_{1},\tilde{U}\right){\left(PX_{1},\tilde{U}\right)}^{T}=\tilde{U}{\tilde{U}}^{T}+PX_{1}X_{1}^{T}P^{T}=X_{1}X_{1}^{T}+X_{2}X_{2}^{T}=XX^{T}=M_{R}M_{R}^{T}.$$

Now we apply a singular value decomposition on ***M****_R_* = ***SDV*** where $S\in O_{n}$, $V\in O_{p}$ and ***D*** is a diagonal matrix with nonincreasing nonnegative diagonal elements. Then, due to (B.2), the singular decomposition of $\left(PX_{1},\tilde{U}\right)$ is $SD\tilde{V}$ for a $\tilde{V}\in O_{p}$. Therefore $\tilde{B}={\tilde{V}}^{T}V$ is the orthogonal matrix satisfies (3.4). □

## Appendix C. Bound on condition (3.7).

To achieve the condition (3.7) we can specify the noise distribution to have large noise values. Let *x_max_* denote the largest possible absolute value of entries in ***X***_1_. Then

$$\lambda_{max}\left(X_{1}X_{1}^{T}\right)={\left\|X_{1}\right\|}_{2}^{2}\leq {\left\|X_{1}\right\|}_{F}^{2}=\sum_{i=1}^{n}\sum_{j=1}^{p_{1}}x_{1,ij}^{2}\leq np_{1}x_{max}^{2}=C_{n},$$

where ‖ · ‖_2_ and ‖ · ‖*_F_* are the operator norm and Frobenius norm respectively. Note that $C_{n}=np_{1}x_{max}^{2}$ is a known constant to the designer of the TM^2^ scheme. Condition (3.5) holds when $\lambda_{min}\left(X_{2}X_{2}^{T}\right)$ exceeds this constant.

We require each data provider to generate a *p*_2_-dimensional random noise vector with i.i.d. Gaussian elements of mean zero and variance *σ*^2^. Assume that *γ* = *p*_2_/*n* > 1, when *n* → ∞, Corollary 13 in Ledoux et al. [2010] provides a probability bound on the $\lambda_{min}\left(X_{2}X_{2}^{T}\right)$ for any *δ* > 0:

$$Pr\left[\lambda_{min}\left(X_{2}X_{2}^{T}\right)\leq {\left(\sqrt{\gamma }-1\right)}^{2}n\sigma^{2}(1-\delta )\right]\leq C_{0}e^{-n\delta^{3/2}/C_{0}},$$

for some constant *C*_0_. The bound on the right side decrease exponentially in *n* so that, for large *n*, it can be made smaller than *ϵ* for a *δ* < 1. Choosing $\sigma^{2}>C_{n}/\left[{\left(\sqrt{\gamma }-1\right)}^{2}n(1-\delta )\right]$ will ensure that (3.7) holds.

## Appendix D. Proof of Theorem 3.8.

### Proof.

We study the posterior density

(D.1) $$\pi_{X_{1}|\left(M_{L},M_{R}\right)}\left(x_{1}|m_{L},m_{R}\right)=\frac{\pi_{\left(X_{1},M_{L},M_{R}\right)}\left(x_{1},m_{L},m_{R}\right)}{\int_{{{}_{S}}_{{}_{X_{1}}}\left(m_{L},m_{R}\right)}\pi_{\left(X_{1},M_{L},M_{R}\right)}\left(x_{1}^{*},m_{L},m_{R}\right)dx_{1}^{*}},$$

and compare it with the prior density $\pi_{X_{1}}\left(x_{1}\right)$ restricted on the support $S_{X_{1}}\left(m_{L},\;M_{R}\right)$.

Recall that the probability densities for ***X***_1_, ***X***_2_, ***A*** and ***B*** at values ***X***_1_ = ***x***_1_, ***X***_2_ = ***x***_2_, ***A*** = ***a*** and ***B*** = ***b*** are denoted respectively as $\pi_{X_{1}}\left(x_{1}\right)$, $\pi_{X_{2}}\left(x_{2}\right)$, *π****_A_***(***a***) and *π****_B_***(***b***). Due to the independence of the generation mechanism of these quantities, their joint density is

(D.2) $$\pi_{\left(X_{1},X_{2},A,B\right)}\left(x_{1},x_{2},a,b\right)=\pi_{X_{1}}\left(x_{1}\right)\pi_{X_{2}}\left(x_{2}\right)\pi_{A}(a)\pi_{B}(b),$$

for $\left(X_{1},X_{2},A,B\right)\in S_{X_{1}}\times S_{X_{2}}\times S_{A}\times S_{B}$.

Since the elements of ***X***_2_ are i.i.d. from the Gaussian distribution *N*(0*, σ*^2^),

$$\pi_{X_{2}}\left(x_{2}\right)=\frac{1}{{\left(\sqrt{2\pi }\sigma \right)}^{np_{2}}}e^{-\frac{\sum_{1\leq i\leq n,1\leq j\leq p_{2}}x_{2,ij}^{2}}{2\sigma^{2}}}=f\left({\left\|x_{2}\right\|}_{F}^{2}\right),$$

where $f(x)=\frac{1}{{\left(\sqrt{2\pi }\sigma \right)}^{np_{2}}}e^{-\frac{x}{2\sigma^{2}}}$ and ${\left\|x_{2}\right\|}_{F}^{2}=\sum_{1\leq i\leq n,1\leq j\leq p_{2}}x_{2,ij}^{2}$ with ‖ · ‖*_F_* denote the Frobenius norm. Thus the joint density becomes

(D.3) $$\pi_{\left(X_{1},X_{2},A,B\right)}\left(x_{1},x_{2},a,b\right)=\pi_{X_{1}}\left(x_{1}\right)f\left({\left\|x_{2}\right\|}_{F}^{2}\right)\pi_{A}(a)\pi_{B}(b).$$

We now plug (D.3) into (D.1) for calculation.

First, we calculate the numerator $\pi_{\left(X_{1},M_{L},M_{R}\right)}\left(x_{1},m_{L},m_{R}\right)$ in (D.1). We denote the restricted sample spaces of random variables ***A*** and ***B*** respectively given knowledge of some other quantities as:

(D.4) $$\begin{array}{l} S_{A}\left(x_{1},m_{L}\right)=\left\{a:ax_{1}=m_{L}\right\},\\ S_{B}\left(x_{1},m_{R}\right)=\left\{b:\exists x_{2}\;\text{such}\;\text{that}\;\left(x_{1},x_{2}\right)b=m_{R}\right\}. \end{array}$$

Notice that given ***A*** = ***a*** and ***M****_L_* = ***m****_L_*, then ***X***_1_ = ***a****^T^****m****_L_*. Also, given ***B*** = ***b*** and ***M****_R_* = ***m****_R_*, then (***X***_1_, ***X***_2_) = ***m****_R_****b****^T^* so that

$${\left\|X_{2}\right\|}_{F}^{2}=trace\left(X_{2}X_{2}^{T}\right)=trace\left[m_{R}b^{T}bm_{R}^{T}-X_{1}X_{1}^{T}\right]=trace\left(m_{R}m_{R}^{T}\right)-trace\left(X_{1}X_{1}^{T}\right).$$

Hence given ***A*** = ***a***, ***M****_L_* = ***m****_L_* and ***B*** = ***b***, we have

$${\left\|X_{2}\right\|}_{F}^{2}=trace\left(m_{R}m_{R}^{T}\right)-trace\left(a^{T}m_{L}m_{L}^{T}a\right)=trace\left(m_{R}m_{R}^{T}\right)-trace\left(m_{L}m_{L}^{T}\right).$$

Then using this and equation (D.3), we have

(D.5) $$\pi_{\left(X_{1},M_{L},M_{B}\right)}\left(x_{1},m_{L},m_{R}\right)\;=\int_{S_{A}\left(x_{1},m_{L}\right)}\{\int_{S_{B}\left(x_{1},m_{R}\right)}\pi_{X_{1}}\left(x_{1}\right)f\left[trace\left(m_{R}m_{R}^{T}\right)-trace\left(m_{L}m_{L}^{T}\right)\right]\pi_{A}(a)\pi_{B}(b)db\}da\;=\pi_{X_{1}}\left(x_{1}\right)f\left[trace\left(m_{R}m_{R}^{T}\right)-trace\left(m_{L}m_{L}^{T}\right)\right]\int_{S_{A}\left(x_{1},m_{L}\right)}\{\int_{S_{B}\left(x_{1},m_{R}\right)}\pi_{A}(a)\pi_{B}(b)db\}da\;=\pi_{X_{1}}\left(x_{1}\right)f\left[trace\left(m_{R}m_{R}^{T}\right)-trace\left(m_{L}m_{L}^{T}\right)\right]\int_{S_{A}\left(x_{1},m_{L}\right)}[\int_{S_{B}\left(x_{1},m_{R}\right)}\pi_{B}(b)db]\pi_{A}(a)da\;=\pi_{X_{1}}\left(x_{1}\right)f\left[trace\left(m_{R}m_{R}^{T}\right)-trace\left(m_{L}m_{L}^{T}\right)\right]\left[\int_{S_{A}\left(x_{1},m_{L}\right)}^{S_{A}\left(x_{1},m_{L}\right)S_{B}\left(x_{1},m_{R}\right)}\pi_{A}(a)da\right]\left[\int_{S_{B}\left(x_{1},m_{R}\right)}\pi_{B}(b)db\right].$$

Now for any pair of ***x***_1_ and $x_{1}^{*}$ that both belongs to $S_{X_{1}}\left(m_{L},\;m_{R}\right)$, there exist (***a***, ***a***^∗^) such that $ax_{1}=m_{L}=a*x_{1}^{*}$. Denote ***A***_0_ = (***a***)^−1^***a***∗. Then $A_{0}x_{1}^{*}={\left(a\right)}^{-1}m_{L}=x_{1}$ and $A_{0}^{-1}x_{1}=x_{1}^{*}$. Hence for any $\tilde{a}\in S_{A}\left(x_{1},m_{L}\right)$ we have

$$\tilde{a}A_{0}x_{1}^{*}=\tilde{a}x_{1}=m_{L},$$

i.e., $\tilde{a}A_{0}\in S_{A}\left(x_{1}^{*},m_{L}\right)$. On the other hand, for any $\bar{a}\in S_{A}\left(x_{1}^{*},m_{L}\right)$, $\bar{a}A_{0}^{-1}x_{1}=\bar{a}x_{1}^{*}=m_{L}$, i.e., $\bar{a}A_{0}^{-1}\in S_{A}\left(x_{1},m_{L}\right)$. Taken together, we have a one-to-one mapping between the two sets $S_{A}\left(x_{1},m_{L}\right)$ and $S_{A}\left(x_{1}^{*},m_{L}\right)$. Particularly,

(D.6) $$S_{A}\left(x_{1}^{*},m_{L}\right)=S_{A}\left(x_{1},m_{L}\right)A_{0}.$$

Hence for uniform density *π****_xA_*** = *π*_0_, (D.6) and Lemma 3.4 implies that

(D.7) $$\int_{S_{A}\left(x_{1},m_{L}\right)}\pi_{A}(a)da=\int_{S_{A}\left(x_{1}^{*},m_{L}\right)}\pi_{A}(a)da.$$

Plug (D.5) and (D.7) into (D.1) and cancel the common factors, we get

(D.8) $$\begin{array}{l} \;\;\;\;\;\;\;\;\;\;\;\;\;\;\;\;\;\pi_{X_{1}|\left(M_{L},M_{R}\right)}\left(x_{1}|m_{L},m_{R}\right)\\ =\frac{\pi_{X_{1}}(x_{1})[\int_{S_{B}\left(x_{1},m_{R}\right)}\pi_{B}(b)db]}{\int_{S_{X_{1}}(m_{L},m_{R})}\pi_{X_{1}}\left(x_{1}^{*}\right)[\int_{S_{B}\left(x_{1}^{*},m_{R}\right)}\pi_{B}(b)db]dx_{1}^{*}}, \end{array}$$

Next, for any pair of ***x***_1_ and $x_{1}^{*}$ that both belongs to $S_{X_{1}}\left(m_{L},\;m_{R}\right)$, there exist (***x***_2_, ***b***) and $\left(x_{2}^{*},b*\right)$ such that $\left(x_{1},x_{2}\right)b=m_{R}=\left(x_{1}^{*},x_{2}^{*}\right)b*$. Let ***B***_0_ = ***b***(***b***∗)^−1^. Then $\left(x_{1},x_{2}\right)B_{0}=\left(x_{1}^{*},x_{2}^{*}\right)$. Similar to (D.6), we have

(D.9) $$S_{B}\left(x_{1},m_{R}\right)=B_{0}S_{B}\left(x_{1}^{*},m_{R}\right).$$

For uniform density *π****_B_*** = *π*_0_, using (D.9), Lemma 3.4 implies that

(D.10) $$\int_{S_{B}\left(x_{1},m_{R}\right)}\pi_{B}(b)db=\int_{S_{B}\left(x_{1}^{*},m_{R}\right)}\pi_{B}(b)db$$

Plug-in (D.10) into equation (D.8), we have

$$\pi_{x_{1}|\left(M_{L},M_{R}\right)}\left(x_{1}|m_{L},m_{R}\right)=\frac{\pi_{X_{1}}\left(x_{1}\right)}{\int_{S_{X_{1}}\left(m_{L},m_{R}\right)}\pi_{X_{1}}\left(x_{1}^{*}\right)dx_{1}^{*}}.$$

□
